# Protective Effects of Salidroside against Carbon Tetrachloride (CCl_4_)-Induced Liver Injury by Initiating Mitochondria to Resist Oxidative Stress in Mice

**DOI:** 10.3390/ijms20133187

**Published:** 2019-06-28

**Authors:** Shi-Yu Lin, Dan Xu, Xia-Xia Du, Chong-Lin Ran, Lu Xu, Shao-Jun Ren, Zi-Ting Tang, Li-Zi Yin, Chang-Liang He, Zhi-Xiang Yuan, Hua-Lin Fu, Xiao-Ling Zhao, Gang Shu

**Affiliations:** 1 Department of Pharmacy, Veterinary Medicine College of Sichuan Agricultural University, Chengdu 611130, China; 2 Department of Animal Science, College of Animal Science and Technology, Sichuan Agricultural University, Chengdu 611130, China

**Keywords:** Salidroside, CCl_4_, liver injury, mitochondria, oxidative stress

## Abstract

The antioxidant effect of salidroside has been proven, but its role in liver injury is poorly understood. In this study, we aimed to evaluate the protective effects and mechanism of salidroside on liver injury induced by carbon tetrachloride (CCl_4_) in vivo. Mice were pretreated with salidroside (60 mg/kg, intraperitoneally injected, i.p.) once per day for 14 consecutive days and then administered with CCl_4_ (15.95 g/kg, i.p.) for 24 h to produce a liver injury model. Salidroside attenuated hepatic transaminase elevation in serum and ameliorated liver steatosis and necrosis, thereby suggesting its protective effect on the liver. Salidroside antagonized CCl_4_-induced toxicity by equilibrating antioxidation system, thereby inhibiting reactive oxygen species accumulation, and restoring mitochondrial structure and function. Salidroside exerts antioxidant and liver-protective effects by selectively inhibiting the activation of genes, including growth arrest and DNA -damage-inducible 45 α (Gadd45a), mitogen-activated protein kinase 7 (Mapk7), and related RAS viral oncogene homolog 2 (Rras2), which induce oxidative stress in the mitogen-activated protein kinase pathway. These results revealed that salidroside can protect the liver from CCl_4_-induced injury by resisting oxidative stress and protecting mitochondrial function.

## 1. Introduction

**Figure 1:**
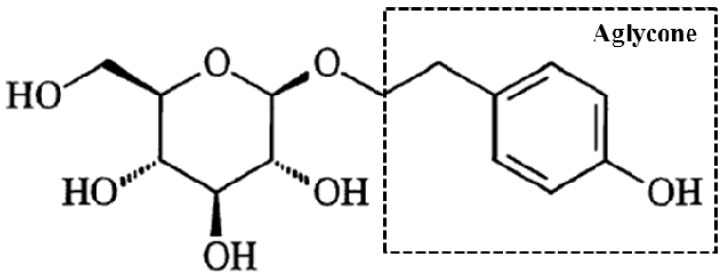
Chemical structural formula of salidroside (p-hydroxyphenethyl-β-D-pyran glucoside, Mr = 300.3). Aglycone denoted with the dotted box is the radical-scavenging active structure of salidroside in vitro.

Salidroside (p-hydroxyphenethyl-β-D-glucoside, Figure 1) is an essence in Tibetan *Rhodiola rosea* L. that is a rare medicinal herb known as “ginseng from plateau” [1]. Salidroside has many pharmacological activities, such as free radical scavenging, cell membrane protection, nervous and cardiovascular system promoting effects, metabolism regulation, immune function improvement, and aging prevention [2,3]. Salidroside is also a safe agent that can decrease the liver enzymes level and suppress inflammatory reactions in the serum to reduce pathological damage in concanavalina-induced liver injury. Salidroside can also modulate apoptosis and autophagy via phosphatidylinositol-3-kinase/serine-threonine protein kinase B pathway in mice [4]. The protective effects of salidroside against furan-induced hepatocyte damage are due to its excellent ability to scavenge free radicals [5]. The radical-scavenging function of salidroside is mostly related to its aglycone in vitro antioxidant activity [6]. Zhang et al. [7] reported that salidroside can alleviate hepatic steatosis in C57BLKS/Leprdb(db/db) mice with type 2 diabetic. This compound can also dose-dependently regulate lipid accumulation, reactive oxygen species (ROS) generation, and inflammasome activation against high-fat diet-induced nonalcoholic fatty liver disease [8,9]. In particular, salidroside has an excellent protective efficacy on liver disease via its antioxidant activities [5,10].

Exogenous chemicals and their toxic metabolites can be transformed or stored in the liver to induce acute or chronic liver injury. Carbon tetrachloride (CCl_4_) is commonly used to establish liver injury models in animals because it induces free radicals and triggers a peroxide chain reaction. Cytochrome P450 converts CCl_4_ to trichloroethane radical (CCl_3_), which combines with O to generate a considerable number of active trichloromethyl peroxyl radicals (CCl_3_O_2_·) with spare O molecules [11,12]. These toxic byproducts induce a chain reaction and lipid peroxidation in phospholipid-rich and membrane-like structures, such as the mitochondria and the endoplasmic reticulum. Membrane permeability breaks when a series of changes, including the disturbance of cell Ca homeostasis, unbalanced antioxidant system, and the formation of covalent bonds between O free radicals and nucleic acids or proteins, occurs [13,14,15,16]. This domino effect leads to cell damage. Inflammatory response activates the neutrophil respiratory burst to produce O free radicals, thereby aggravating oxidative stress and liver damage [17]. These initiating factors can further activate the Toll-like receptor, mitogen-activated protein kinase (MAPK), and other signal pathways that set off mitochondrial permeability transition pore opening [18]. Consequently, hepatocyte apoptosis and necrosis are activated and may even progress in liver cancer.

In this study, we established a mouse model for acute liver injury induced by CCl_4_. Diammonium glycyrrhizinate (DG), which is a third-generation licorice extract, is a prescription drug for liver disease with some protective effects against liver damage induced by chemicals, such as CCl_4_ [19]. DG was used as a positive control in the present study. We detected liver index and hepatic transaminases in the serum and analyzed liver histopathological changes to determine the liver-protective effect preliminarily. Biochemical indices including liver antioxidation, ROS status, and mitochondrial membrane potential (MMP) were determined in the liver to verify the effects of salidroside by equilibrating antioxidation system and restoring mitochondrial structure and function. Genes, including growth arrest and DNA damage-inducible 45 α (Gadd45a), mitogen-activated protein kinase 7 (Mapk7), and related RAS viral oncogene homolog 2 (Rras2), are related to oxidative stress in the MAPK pathway and may be selectively inhibited. This study aimed to clarify the underlying protective mechanism of salidroside on CCl_4_-liver injury mice by initiating mitochondria to resist oxidative stress.

## 2. Results

### 2.1. Salidroside Antagonized CCl_4_-Induced Hepatotoxicity in Mice

**Figure 2:**
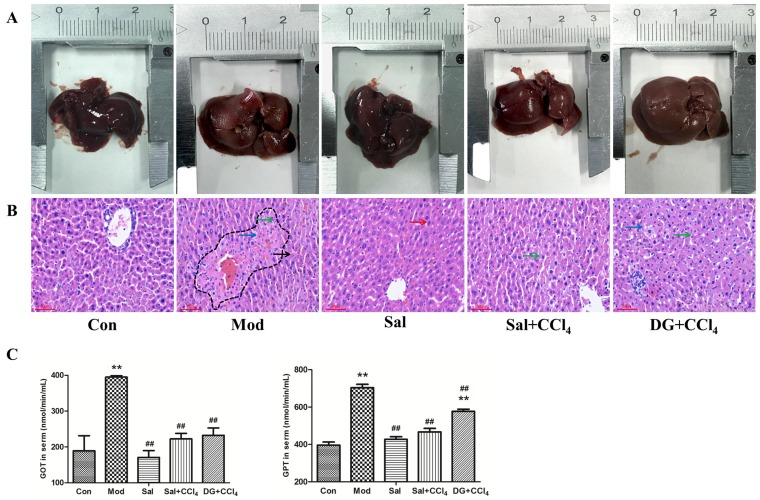
Salidroside attenuated carbon tetrachloride (CCl_4_)-induced liver steatosis and decreased hepatic transaminases in mice (*n* = 6). (**A**) Macroscopic characteristic of livers in control mice, model mice induced by CCl_4_, and mice treated with single salidroside, salidroside+CCl_4_, and diammonium glycyrrhizinate+CCl_4_ (Con, Mod, Sal, Sal+CCl_4_, and DG+CCl_4_, respectively). Livers in the Con and Sal groups had normal gross morphology. However, the livers of mice in Mod, Sal+CCl_4_, and DG+CCl_4_ groups showed increased volumes, decreased glossiness, and smoothness. The most severe and slightest lesions were observed in the Mod and the Sal+CCl_4_ groups, respectively. (**B**) Pathologic histology of livers in different groups. Mild steatosis can be observed in the liver of the Con group, and the Sal group showed a normal structure. Histopathological changes showed the fatty degeneration and necrosis of hepatocytes and infiltration of inflammatory cells, which were more and increasingly mild in the Mod, Sal+CCl_4_, and DG+CCl_4_ groups. Red arrows marked normal hepatocytes, green arrows marked cell nucleus pyknosis, black arrows marked cell nucleus broken, blue arrows marked vacuoles caused by steatosis, and dotted line denoted necrotic area. Magnification: 200×. (**C**) GOT and GPT activities in the serum of different groups. CCl_4_ treatment increased the serum GOT and GPT levels (*p* < 0.05). Salidroside reduced the CCl_4_-induced transaminase, and DG only alleviated GOT condition significantly (*p* < 0.05). The single-treatment of salidroside had no side-effect. *p* < 0.05 was considered statistically significant. **p < 0.05 (groups compared with the Con group) and ##*p* < 0.05 (groups compared with the Mod group). (GOT: glutamic oxalacetic transaminase; GPT: glutamic-pyruvic transaminase).

The difference in the body weight and liver index among groups was insignificant (*p* > 0.05). The macroscopic characteristics of livers were shown in Figure 2A. The livers in the control (Con) and salidroside (Sal, received only salidroside) groups were normal with a red-brown, bright, and damp surface. Livers were slightly swelled with a tense capsular, blunt edge, and low-gloss surface with the typical lesions of legible yellowish-brown spot-like steatosis due to CCl_4_. Salidroside and DG alleviated the condition of livers to be slightly less glossy and showed mild steatosis compared with that of the model (Mod, CCl_4_ only) group.

Histopathological (Figure 2B) examinations showed the presence of regularly lined hepatic cords and hepatocytes with few mild steatosis possibly because of the slight stimulation of colza oil in the liver of the Con group. In the Sal group the livers were intact, thereby showing as neatly arranged hepatic cords, which were better than that of Con. CCl_4_ caused moderate hepatocyte steatosis (many lipid droplet vacuoles with varying sizes), extensive necrosis (nuclear fragments resulting from nuclear concentration, fragmentation, or dissolution) around the central vein, and inflammatory cell infiltration. Salidroside and DG can improve the status of hepatocytes, particularly salidroside. The moderate steatosis and scattered necrosis of hepatocytes and infiltration of inflammatory cells were observed in salidroside prevention group (Sal+CCl_4_, received salidroside and CCl_4_), whereas more severe steatosis was found in the DG prevention group (DG+CCl_4_, received with DG and CCl_4_) than that in the Sal+CCl_4_ group.

The transaminase activities in the serum (Figure 2C) suggested a successful CCl_4_-model and effective liver protection of salidroside. Glutamic oxalacetic transaminase (GOT) and glutamic pyruvic transaminase (GPT) activities are the two important reliable markers of liver function. The compound can normalize the nearly doubled GOT and GPT activities induced by CCl_4_, whereas DG was effective only in increasing GOT. The use of salidroside did not show side effects on the liver.

### 2.2. Salidroside Balanced Antioxidant System against Oxidative Stress

**Figure 3:**
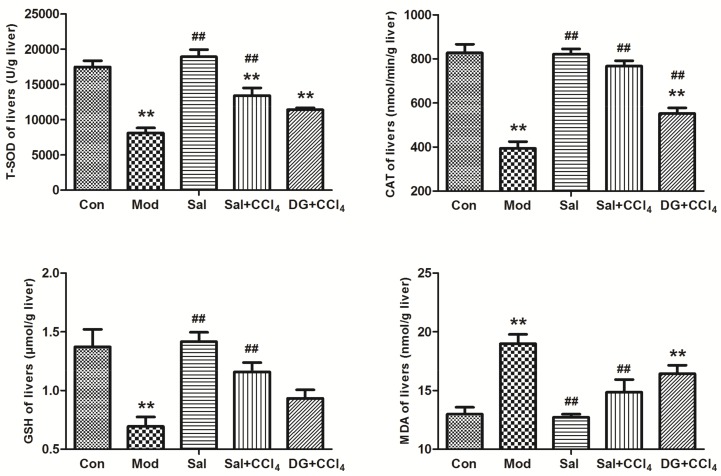
Salidroside balanced liver antioxidant system for oxidative stress resistance in mice. The vital antioxidases, namely, TSOD and CAT, nonenzymatic antioxidant GSH, and lipid peroxidative product MDA levels in the liver of different groups, showed that the model mice were induced by CCl_4_ and mice treated with single salidroside, salidroside+CCl_4_, and diammonium glycyrrhizinate+CCl_4_ (Con, Mod, Sal, Sal+CCl_4_, and DG+CCl_4_ groups, respectively). In the Mod group, the SOD, CAT, and GSH levels were significantly reduced, but the MDA level increased (*p* < 0.05). The condition can be reversed in the Sal+CCl_4_ and DG+CCl_4_ groups compared with that in the Mod group (*p* < 0.05), but the TSOD level in the Sal+CCl_4_ group and CAT/GSH level in the DG+CCl_4_ group showed a weak remission_._ The difference between the Sal and Con groups was insignificant (*p* > 0.05). *p* < 0.05 was considered to be statistically significant. **p < 0.05 (groups compared with the Con group) and ##*p* < 0.05 (groups compared with the Mod group). (CCl_4_: carbon tetrachloride; TSOD: total superoxide dismutase; CAT: catalase; GSH: glutathione-SH; MDA: malonaldehyde).

Figure 3 showed the liver antioxidant-related biochemistry, which revealed the effective antioxidant activities of salidroside. Total superoxide dismutase (TSOD) and catalase (CAT) are the main antioxidant enzymes, and glutathione-SH (GSH) is an important nonenzymatic reductant in the body. These enzymes participate in ROS scavenging. As shown in Figure 3, these levels were reduced observably in Mod. Salidroside or DG treatment can reversely enhance CCl_4_ levels, but TSOD level had a weak remission in Sal+CCl_4_ (*p* < 0.05). Malonaldehyde (MDA) is a marker of free radical-mediated lipid peroxidation damage. The MDA level in the Sal+CCl_4_ group decreased compared with that in the Con group. In the DG+CCl_4_ group, TSOD level was significantly lower, and MDA level was significantly higher than those in Con (*p* < 0.05). DG showed a weak balancing ability and a slight TSOD and CAT level recovery. The difference between the Sal and Con groups was insignificant. In summary, salidroside played an outstanding anti-oxidative role in CCl_4_-model mice and possessed the greater liver preservation ability than commercial DG.

### 2.3. Salidroside Protected Mitochondria

**Figure 4:**
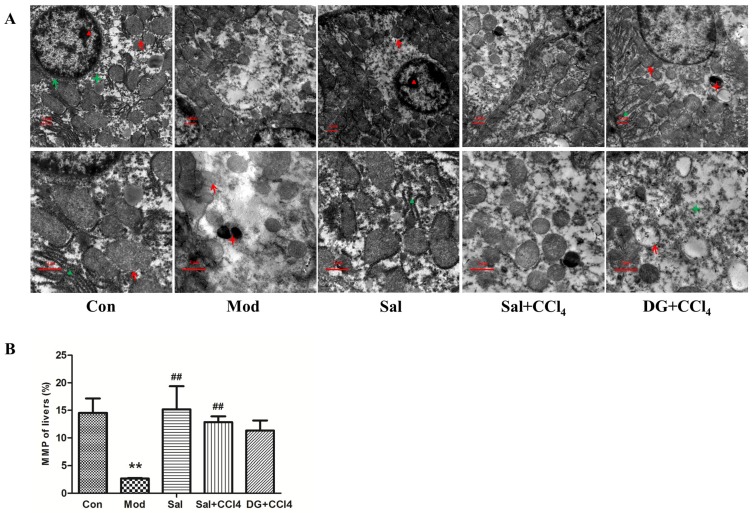
Salidroside protected liver ultrastructure, especially mitochondria in mice. (**A**) Conditions of liver ultrastructure in different groups: model mice induced by CCl_4_ and mice treated with single salidroside, salidroside+CCl_4_, and diammonium glycyrrhizinate+CCl_4_ (Con, Mod, Sal, Sal+CCl_4_, and DG+CCl_4_, respectively). In the Mod group, CCl_4_ induced ultrastructural damage with decreased chromatin and organelles, partly dissolved ridges and membrane of swollen mitochondria, and increased secondary lysosomes. Salidroside and DG can remarkably alleviate ultrastructural damage, but the former was better than the latter. Single salidroside treatment had no side effect on liver ultrastructure. Red and green arrows marked mitochondria and hepatocytes, respectively. Red and green triangles marked nucleolus and endoplasmic reticulum, respectively. Red and green stars marked secondary lysosome and glycogen, respectively. Magnification: 6000× in the first row, 12000× in the second row. (**B**) Status of liver MMP in different groups. CCl_4_ caused excessively low MMP, whereas salidroside effectively normalized the MMP level and even performed better in terms of the overall liver MMP protection ability than that of DG. *p* < 0.05 was considered statistically significant. **p < 0.05 (groups compared with the Con group) and ##*p* < 0.05 (groups compared with the Mod group). (CCl_4_: carbon tetrachloride; MMP: mitochondrial membrane potential).

The liver ultrastructure was presented in Figure 4A. Abundant mitochondria with clear membrane and aligned ridges, rough endoplasmic reticulum, glycogen, and few lysosomes were found in the liver cytoplasm of the Con group. The structures of these abundant organelles in the cytoplasm of Sal were normal. CCl_4_ decreased the number of chromatins in the cell nucleus, thereby resulting in decreased electron density, decreased number of organelles, and swollen mitochondria with partly dissolved ridges and membrane and leading to local vacancy. The number of secondary lysosomes that had current or undergone digestion also increased. In the Sal+CCl_4_ group, the bilayer nuclear membrane structure was clear, the mitochondria slightly decreased in number and were slightly swollen with intact ridges, and the electron density negligibly decreased compared with those of the Con group. DG remarkably alleviated lesions compared with those in the Mod group. However, some swollen mitochondria, dilated rough endoplasmic reticulum, and secondary lysosomes were still observed in the DG+CCl_4_ group.

Mitochondria are important energy-producing organelles in cells and are also involved in cell apoptosis. The electrochemical potential energy is stored in the inner mitochondrial membrane to form MMP. Figure 4B showed that CCl_4_ caused excessively low MMP, whereas salidroside effectively normalized MMP level and even performed better overall liver MMP protection ability than DG.

### 2.4. Mitochondria Decreased ROS Generation with Salidroside

**Figure 5:**
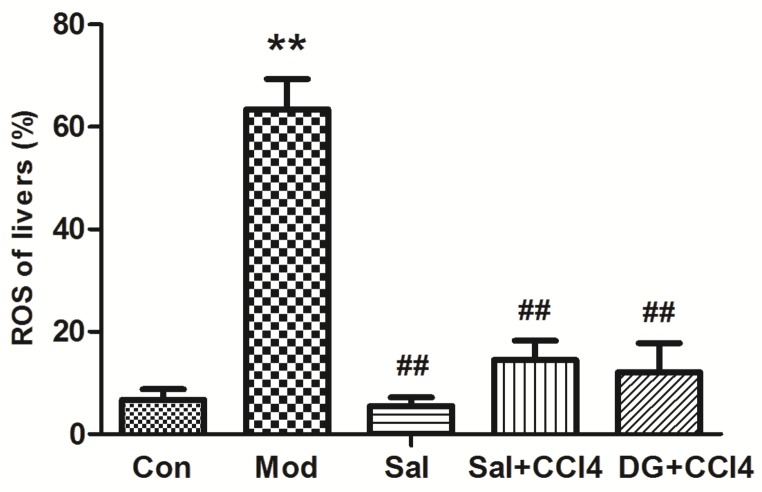
Salidroside inhibited liver reactive oxygen species (ROS) generations in the mice of different groups, that is, model mice induced by CCl_4_ and mice treated with single salidroside, salidroside+CCl_4_, and diammonium glycyrrhizinate+CCl_4_ (Con, Mod, Sal, Sal+CCl_4_, and DG+CCl_4_ groups, respectively). CCl_4_ caused excessively high ROS, whereas salidroside and DG effectively normalized the ROS levels. *p* < 0.05 is statistically significant. **p < 0.05 (groups compared with the Con group) and ##*p* < 0.05 (groups compared with the Mod group). (CCl_4_: carbon tetrachloride).

During normal mitochondrial respiration reaction to generate energy, a considerable number of electrons are transferred for adenosine triphosphate and few are reduced to form ROS [20]. The structure, function, metabolism, and energy production of mitochondria are affected after CCl_4_ enters the hepatocytes and causes the lipid dissolution of mitochondrial membranes [21]. Mitochondrial dysfunction leads to improper electron transportation, which causes additional ROS generation. When excessive ROS is produced in the mitochondria, the antioxidant balance is broken. Afterward, DNA, protein, and the biological membrane are attacked by hazardous ROS. Finally, hepatocyte degeneration and necrosis are observed, thereby inducing oxidative damage and decreased organ function [22]. Our study showed in Figure 5 that the ROS level in the CCl_4_-model was higher than that in the Con group. Conversely, salidroside or DG with/without CCl_4_ can maintain a normal ROS level effectively.

### 2.5. Oxidative Stress-Related Genes Gadd45a, Mapk7, and Rras2 Were Involved in Protective Effects of Salidroside against Liver Injury

**Figure 6:**
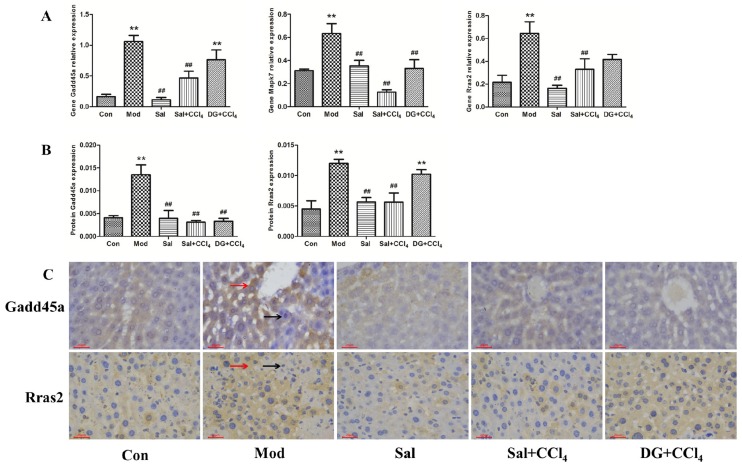
Salidroside protected against carbon tetrachloride (CCl_4_)-induced liver injury by downregulating oxidative stress-related gene Gadd45a, Mapk7, and Rras2. (**A**) Relative expression levels of Gadd45a, Mapk7, and Rras2 genes in different groups, that is, model mice induced by CCl_4_, and mice treated with single salidroside, salidroside+CCl_4_, and diammonium glycyrrhizinate+CCl_4_ (Con, Mod, Sal, Sal+CCl_4_, and DG+CCl_4_ groups, respectively). *p* < 0.05 was considered to be statistically significant. **p < 0.05 (groups compared with the Con group) and ##*p* < 0.05 (groups compared with the Mod group). (**B**) Optical density statistics of Gadd45a and Rras2 proteins expression levels in different groups. (**C**) Immunohistochemical staining of Gadd45a and Rras2 proteins in different groups. Gadd45a was expressed and distributed in the cytoplasm surrounding the centrilobular veins of the liver in the CCl_4_ group and seldom expressed in other groups. Rras2 protein was highly expressed in a sheet in CCl_4_ and DG+CCl_4_ groups and partially expressed in the hepatocyte of other groups. Brown-yellow or brown substances expressed in the cytoplasm were positive proteins (red arrows). Cell nucleus was stained blue (black arrows). Magnification: 200×. (Gadd45a: growth arrest and DNA damage-inducible 45 α; Mapk7: mitogen-activated protein kinase 7; Rras2: related RAS viral oncogene homolog 2).

The mRNA levels of the oxidative stress-related gene Gadd45a, Mapk7, and Rras2 were significantly elevated in the livers of mice in the Mod group compared with those in the corresponding Con groups (Figure 6A, *p* < 0.05). The mRNA expression levels of these genes in Sal+CCl_4_ showed 0.56-, 0.80-, and 0.49-fold decreases compared to those in Mod, and DG only affected Mapk7 downregulation. Salidroside did not show side effects on these genes. In terms of corresponding proteins (Figure 6B), the expression levels of Gadd45a and Rras2 in the Mod group showed 2.67- and 3.31-fold growth, respectively. Salidroside can effectively decrease the elevated protein levels induced by CCl_4_ to be close to that in the Con group. The accommodation effect of DG was weaker than that of salidroside, and only Gadd45a protein expression was downregulated. Figure 6C showed the specific condition of immunohistochemical staining in the livers of experimental animals. Gadd45a and Rras2 proteins were expressed in the cytoplasm where brown or yellow substances were found. Gadd45a protein was expressed in the hepatocyte surrounding the centrilobular veins of the liver and high expression in the CCl_4_-model and low expression in the other groups were observed in Figure 6B. Rras2 protein was expressed abundantly in a sheet in the hepatocytes of the CCl_4_ and DG+CCl_4_ groups and expressed locally in other groups.

## 3. Discussion

Our findings showed that salidroside can protect the liver against CCl_4_-induced injury in mice, and the mechanisms may be related to the suppression of oxidative stress and protection of mitochondrial function via the downregulation of the Gadd45a, Makp7, and Rras2 mRNA expression levels.

The liver biological functions, including blood clotting factor generation, bile acid secretion, destruction of bacteria in the blood, and detoxification, are complex. Liver injury may be triggered by microbes, drugs, xenobiotics, and metabolites in the liver [23]. Salidroside, as one of the effective components of *Rhodiola rosea* L., can protect the liver from injury in rats via its antioxidative stress and antiapoptotic functions [24,25]. In the present study, histological analysis and anatomical observations indicated that salidroside can attenuate CCl_4_-induced liver injury. GOT and GPT, as marker enzymes, are also important indicators of liver injury. GOT mainly exists in the hepatocytes; when 1% of hepatocytes are damaged, GOT leaks into the blood at a level approximately 3000-fold higher than that in a cell [26,27]. Salidroside can ease the release of liver transaminase from damaged hepatocytes to protect liver against injury, which is consistent with our results [28]. The present study showed that salidroside can antagonize CCl_4_-induced increase GOT and GPT levels to reach normal stage.

The CCl_4_-induced liver injury model is a classic liver injury model. When CCl_4_ enters an animal’s body, it is activated by the liver cytochrome P450 to generate the free radicals CCl_3_·and CCl_3_O_2_· [29]. These free radicals cause the peroxidization of the fatty acids of the mitochondrial membrane and impair the integrity and stability of the mitochondrial structure, which leads to mitochondrial dysfunction. Once the mitochondrial permeability transition pore is opened, the mitochondria will swell, thereby resulting in low MMP, which is a sensitive index used to evaluate mitochondrial function [30,31,32]. Mitochondrial dysfunction causes uncoupled oxidative phosphorylation in energy respiration. A considerable number of electrons leak out from the uncoupled electron transport chain [30,31]. These electrons are reduced to O molecules to form ROS. The high ROS level initiates lipid peroxidation, depletes free radical scavengers, collapses the body’s antioxidant system, and leads to explosive oxidative stress in the body [32]. Therefore, the structural and functional integrity of the mitochondria is important in resisting oxidative damage and maintaining the normal function of cells. Our findings showed that salidroside can effectively protect the integrity of liver mitochondria, maintain MMP, and reduce ROS formation caused by CCl_4_-induced cytotoxicity.

Understanding the mechanism underlying protective effect of salidroside against CCl_4_-induced liver injury is limited to date. CCl_4_ induces severe liver damage by increasing ROS, which leads to necrosis and apoptosis. Thus, the direct reduction of ROS levels and inhibition of the CCl_4_-induced oxidative chain reaction are critical mechanisms of preventing CCl_4_-induced acute liver injury [33]. Salidroside can increase the main antioxidant enzymes or compound T-SOD, CAT, and GSH levels, which contribute to MDA and ROS scavenging, thereby further protecting the membrane structure from oxidative stress injury [24,28,34], This result is consistent with our results. ROS is an important secondary messenger in the cell that mediates cell survival and death by activating MAPKs [35]. Therefore, the downstream gene Gadd45a, Mapk7, and Rras2 of MAPK were studied to elucidate the molecular mechanism of salidroside. Gadd45a can be activated in oxidative stress, thereby triggering cellular stress response, controlling the G2/M phase of cell cycle, causing imbalance between cell proliferation and apoptosis, finally leading to cell injury and even death [36,37]. The Gadd45a expression level increases in the early stage of ischemia-reperfusion of the heart, lung, liver, and other vital organs, but decreased when irreversible damage occurs at the late stage of liver injury [38,39]. This phenomenon may explain the low Gadd45a protein expression in DG+CCl_4_ groups, although its gene expression in the DG+CCl_4_ group is high. Mapk7 is expressed in most cells and can be activated by many stimulating factors, such as oxidative stress, mechanical stimulation, and hypertonic environment [40]. Our present study showed that Mapk7 protein expression were lower in the Sal+CCl_4_ group, thereby suggesting that salidroside can resist oxidative damage induced by CCl_4_ in the liver. High ROS accumulation levels can be accompanied by Ras overactivation, while Rras2 is a key subgene in the Ras family [41]. The low protein expression of the Rras2 protein in the Sal+CCl_4_ group also suggested that salidroside can protect the liver from oxidative damage induced by CCl_4_. Previous studies showed that salidroside can resist oxidative stress to protect organs or cells by interfering with the MAPK pathways [42,43,44]. Salidroside protects rat liver against ischemia/reperfusion injury by regulating GSK-3β/Nrf2-dependent antioxidant response and mitochondrial permeability transition [45]. In the present study, salidroside can protect the liver against CCl_4_-induced injury by enhancing antioxidant system function, antagonizing liver mitochondrial dysfunction to suppress ROS generation, and downregulating MAPKs. Hence, salidroside has a better effect against CCl_4_-induced injury than DG.

DG can ease the symptoms of chronic viral hepatitis, and it reduces serum transaminase and bilirubin levels [46]. DG is often used in clinical chronic viral hepatitis [47]. However, the liver-protective effects of DG were not observed in this experiment because DG may not have antioxygenic properties. On the contrary, the present study demonstrated that salidroside has antioxidant functions. Salidroside pre-administration played a preventive and health care role, thereby enabling hepatocytes to respond effectively to toxic damage.

In the present study, salidroside can antagonize CCl_4_-induced toxicity by equilibrating antioxidation system, inhibiting ROS accumulation, and restoring mitochondrial structure and function. The antioxidant and liver-protective effects of salidroside were exerted by selectively inhibiting Gadd45a, Mapk7, and Rras2, which are related to oxidative stress in the MAPK pathway. Further studies on salidroside in the future in liver protection are needed. The results of this study showed that salidroside may have therapeutic potential for liver injury.

## 4. Materials and Methods

### 4.1. Mice

Eight-week-old male (18–22 g) Kunming mice free of specified pathogens were purchased from Chengdu Da Shuo Experimental Animal Co., Ltd. (Chengdu, China). No sex association was found on the results of this study. The mice were housed in a controlled environment at 20 ± 2 °C and 55% humidity under a 12 h dark–light cycle. The trial was approved by the Experimental Animals Research Ethics Committee of the Sichuan Agricultural University (21 Jul 2018). Written informed consent was obtained from all participants.

### 4.2. Chemicals

Salidroside with the purity of > 98% was examined via HPLC (Biopurify Phytochemicals Co., Chengdu, China). Other chemicals used were DG (Chia Tai Tianqing, Lianyungang, China), colza oil (Huida Grain and Oil Co., Mianyang, China), and CCl_4_ (Chron Chemicals Co., Chengdu, China). Microdetermination biochemical kits (Cominbiot, Suzhou, China), total ROS assay kit, TRIzol reagent (Invitrogen, China), MMP detection JC-1 kit (BD Bioscience, USA), reverse transcription system (TaKaRa, Japan), and SP9001 histochemical antibody kit (ZSGB-Bio, Beijing, China) were also utilized. Ab5218-050 anti-Gadd45a and Ab51198-050 anti-Rras2 were applied (Multi-Science, Shanghai, China).

### 4.3. Experimental Procedure

A total of 60 mice were selected and randomly divided into five groups with two repetitions containing six mice each for dosing: group A, control; group B, CCl_4_ model; group C, salidroside control (treatment with salidroside 60 mg/kg without CCl_4_ injection); groups D and E were CCl_4_ and salidroside (60 mg/kg) or DG (11.25 mg/kg)-treated mice, respectively.

After acclimation for a week, prophylactic therapy was performed with a protocol according to our pilot experiments. In brief, groups A (vehicle control) and B (CCl_4_ model) received saline, groups C (drug control) and D received salidroside (60 mg/kg), and group E (positive control) was treated with DG (11.25 mg/kg). The treatments were administered once a day for 14 consecutive days. At 2 h after the last drug administration for acute liver injury model, groups B, D, and E received 1% CCl_4_ (15.95 g/kg) diluted in colza oil, whereas groups A and C were given colza oil instead of 1% CCl_4_. All animals were sacrificed under anesthesia after 24 h of modeling. Intraperitoneal injection was the only route of administration for our study. All mice were weighed once every 3 days for accurate dosage and data recording. The increase in in body weight was calculated, as follows (final weight − initial weight)/initial weight.

### 4.4. Organ Index and Serum Biochemistry

Mice livers were weighed immediately after extraction for organ index according to liver weight/mice weight. Blood was obtained from the retrobulbar plexus of anesthetic mice for subsequent serum biochemistry analysis. Blood samples were stored for 2 h at room temperature, and then centrifuged for 10 min at 3500 r/min to isolate the serum, which was kept at −80 °C until use. GPT and GOT levels were measured strictly in accordance with the instructions from the biochemical kits.

### 4.5. Pathologic Histology Observation

Liver samples were fixed in 4% (wt/vol) buffered paraformaldehyde for 24 h. Trimmed samples were dehydrated in embedding cassettes (75% ethanol for 6 h, 85% ethanol for 10 h, 95% ethanol for 4 h, ethyl alcohol I for 2 h, and ethyl alcohol II for 2 h), cleared (xylene I for 20 min and xylene II for 15 min), immersed in paraffin for 3 h, and finally embedded. The samples were cut into 5 µm slices by using a RM2235 microtome (Leica, Germany), flattened onto glass slides in warm water, and then baked at 60 °C for at least 2 h. After dewaxing with xylene, the sections were flushed in water for 20 min, stained with hematoxylin (Thermo, USA) for 30 min, flushed again, differentiated with hydrochloric ethanol, and finally stained with eosin (Thermo, USA) for 5 min. After the sample was dehydrated and cleared again, the samples were sealed with neutral resin size. Liver steatosis and necrosis were observed under a CX22 microscope (Olympus, Japan), and DM1000 microimaging system (Leica, Germany) was used to capture images.

### 4.6. Liver Antioxidant-related Biochemistry

In brief, 3 g liver was placed into a glass homogenizer with 0.9% saline to homogenate at 4 °C. The homogenate was prepared in an ice bath and centrifuged for 10 min at 3500 r·min^−1^. The supernatant was diluted with saline at 1:9 for a subsequent test. The TSOD, CAT, GSH, and MDA levels were subsequently determined according to the instructions of the biochemical kits purchased.

### 4.7. ROS and MMP

Liver samples were cut, centrifuged, and suspended to 1 × 10^6^ cell/L for the following test preparation. The cells were exposed for 15 min at 37 °C with DCFH-DA (total ROS assay kit) in the dark, which was oxidized by superoxide anion into dichlorofluorescein to emit green fluorescence. ROS production was determined using a flow cytometer (Bio-Rad, USA) at 480 nm excitation and 525 nm emission. Prepared cells were incubated for 15 min at 37 °C with JC-1, immediately followed by flow cytometry analysis with 488 nm of excitation and of 590 nm emission for MMP detection.

### 4.8. Ultrastructure Observations

The 1 mm × 1 mm × 1 mm trimmed liver piece was fixed in 2.5% glutaraldehyde (pH = 7.4, 4 °C) for 24 h. The samples were fixed again with OsO_4_ for 2 h and dehydrated with 30% (20 min), 50% (20 min), 70% (25 min), 80% (30 min), 90% (30 min), 100% I (40 min), and 100% II (40 min) acetone. The samples were soaked in epoxy resin acetone solution at 40 °C for 2 h and finally embedded in epoxy resin. Microtome was used to slice the trimmed tissue blocks to 80 nm slices. The sections were double stained with lead citrate and uranyl acetate solution for 15 min. Ultrastructural pathological damage was observed by HT7700 transmission electron microscopy (Hitachi, Japan) and photographed with GANTAN830.10W CCD camera (Hitachi, Japan).

### 4.9. Fluorescence Real-Time Quantification PCR (QRT-PCR)

Liver blood was washed with prefrozen DEPC water and immediately frozen in liquid nitrogen for storage. Approximately 60 mg preserved liver was ground thoroughly with liquid nitrogen in a precooled mortar. Total RNA was extracted with TRIzol. RNA concentration was determined by a nucleic acid protein analyzer, of which the D260/D280 range eligible for reverse transcription was 1.8–2.0. cDNA was stored at −80 °C.

According to the specific steps of SYBR Green Remix Ex Taq^TM^ kit specification of TaKaRa and with β-actin as the internal reference, QRT-PCR was used to detect the expression levels of the genes Gadd45a, Mapk7, and Rras2, and the 2^−ΔΔCT^ method was used to calculate the relative expression levels of these genes. All primers (Table 1) were designed using Premier 5 (PREMIER Biosoft International, USA) and synthesized by Chengke BioTech Co., Ltd.

### 4.10. Immunohistochemistry

The previous paraffin-embedded tissues were cut into 4 μm slides and immunostained by using commercially available SP9001 histochemical antibody kit. The Gadd45a and Rras2 protein expression levels in the liver were detected via immunohistochemical localization with corresponding Rabbit mAb. The deparaffinized slides were placed in citrate buffer (pH = 6.0) for antigen repair, soaked with 30% H_2_O_2_ for 10 min to inactivate endogenous enzymes, and finally sealed and added to 100 μL of goat serum for 15 min. The serum was poured without washing. The samples were incubated overnight at 4 °C with 100 μL of primary antibodies against Gadd45a and Rras2 (rabbit anti-mouse, 1:100), rewarmed at 37 °C for 1 h before the biotinylated secondary antibody IgG was introduced (goat anti-rabbit, 1:200), and streptavidin-peroxidase conjugate was added for 15 min in a moist chamber. The slides were colored using fresh DAB within a controlled reaction time of 5 to 30 min under a microscope. Hematoxylin was used for 20 s of counterstaining. Then, the slides were differentiated, rinsed to blue, dehydrated, cleared, and sealed. PBS was used as a negative control for the primary antibody. Positive protein expression was observed under a microscope and recorded using a microscopic imaging system (the images of three typical positive regions were captured). Optical density measurements were analyzed by Image Pro Plus 6.0 software.

### 4.11. Statistical Analysis

After distribution normality and homoscedasticity testing, the data were analyzed with Tukey HSD multiple range test by using JMP 10 statistical software (SAS, USA) and expressed as mean ± SE. Differences were considered statistically significant at *p* < 0.05.

## 5. Conclusions

CCl_4_ can destroy the mitochondrial structure, reduce MMP, and promote ROS production by enhancing lipid peroxidation and reducing related antioxidants, thereby resulting in oxidative damage to the mitochondria in hepatocytes. However, salidroside can effectively protect the structure and function of the mitochondria, improve antioxidant activity in vivo, and regulate the Gadd45a, Makp7, and Rras2 expression levels, thereby suggesting its antioxidant role. Specific signal pathways involved in these processes must be further studied, especially the mitochondrial apoptotic pathway.

## Figures and Tables

**Table 1:** Primers.

Gene	Forward Primers, 5′–3′	Reverse Primers, 5′–3′
β-Actin	GCCCTGAGGCTCTTTTCCA	GTTGGCATAGAGGTCTTTACGGAT
Gadd45a	GCAGAGCAGAAGACCGAAAG	TAACAGAACGCACGGATGAG
Mapk7	TGTGACCAATGCCAAACGG	GCGGCTGTGAAGAGTGAATGA
Rras2	CAGAGTAAAGGACCGTGATGAG	TGGTTCTGGTGAAGGAGGG
